# Laceration of the Penis

**Published:** 1878-08-20

**Authors:** A. A. Lyon

**Affiliations:** Shreveport, La.


### LACERATION OF THE PENIS.

By A. A. Lyon, M.D., Shreveport, La.

Not long since I was called in great haste to see Willie B., a boy about seven years of age, who, in the performance of his childish pranks, had severely lacerated his penis. The accident occurred in this wise: The little fellow had fastened an ordinary trace-chain at either extremity, to the posts of a wide doorway; the slack of this chain was in the center, supported by a pole or stick into the upper end of which was driven a nail, and this thrust through one of the links, in its application not unlike that of a prop to a clothes line. He leaped astride this chain near its juncture with the prop, and was engaging in a variety of evolutions, which he denominated "playing circus. At an unfortunate moment a fold of the skin investing his penis was caught between the link and the nail, inflicting severe pain and causing him to fall. In this fall the laceration occurred. Upon examination fully three-fourths of the organ was found to be completely denuded. Posteriorly the skin was stripped from a point midway between the scrotum and prepuce forward to the margin of the latter, thence backward anteriorly to the pubis, the separated skin at this point hanging by a narrow strip or pedicle, not wider than a large brown straw. Imagine, if you choose, the finger of a kid glove upon the hand torn from the middle of the palmer aspect of the enclosed finger forward over the end back to the knuckle hanging there only by a shred, leaving three-fourths of the finger bare, and you have an illustration of the case before you.

As seen from above the organ was in its entire length wholly deprived of its natural covering and looked as red and rs raw as a flayed beef. In this aspect of the case the following methods of treatment were suggested. Either, first, to replace and co-aptate the torn skin and stitch it back as accurately as possible, relying upon the little dormal isthmus above alluded to to supply the requisite pabulum for the adhesive and healing processes. Second, to remove the pendulous fragment and trust to superficial granulation without further operation; and third, to incise the foreskin, retract its margin and attach the same by sutures to the remaining sound skin.

The first plan seemed quite feasible, and in all probability would have proven satisfactory but was not adopted for the reason that in case of failure, in this way, to obtain reunion, the parts, after the experiment, would not be in so favorable a condition for another operation as they were at that time.

The second would have been really unsurgical, and was not seriously considered, though it likewise might have terminated favorably.

The plan last named was the one therefore adopted. The operation was the simplest possible: The patient was anæsthetized by my friend, Dr. A. R. Booth, who kindly assisted me on the occasion, and the detached and pendent portion of skin was cut away with a pair of scissors. With a sharp bistoury I then completely divided, superiorly, the foreskin, (which was, as usual, quite redundant), from its margin to the corona glandis, and below and nearly opposite a similar incision contiguous to the frenum, yet taking care to avoid injuring it. The divided prepuce was then carried back and stitched by five or six interrupted sutures to the sound skin behind it. The sutures were made with ordinary surgeon's silk, though for some reasons it might have been best to have employed silver wire, yet the silk was more convenient and is moreover, in some respects preferable, in a case of this kind. In the present instance it answerd well.

The operation, of course, exposed the glands, and constituted practical circumcision.

The after-treatment was as simple as the operation. The organ was enveloped in a little greasy rag, and over this a miniature roller bandage was applied, as a support, and to control the swelling. For a day or two a cold water dressing was used, and afterwards a simple application of carbolized cerate, in conjunction with a mixture of equal parts of baselicon ointment and sweet oil, using one or the other as circumstances seemed to require.

On the fifth and sixth days I removed the sutures. From the beginning the case went on rapidly and without an untoward symptom to complete recovery.

From the health and youth of the subject, and the known reparative powers of the penis, I felt sure the case would terminate favorably. Yet was apprehensive, and so expressed myself to the parents, that there would be more or less deformity, and in the condition of erection, probably inconvenience resulting from the cicatrix, that might in future require another little operation to relieve.

In ten or twelve days the little fellow was up, out and playing as usual, though he expressed his purpose, in future, to eschew all acrobatic circus performances. He passed from my observation till very recently, when I again examined him, and was almost surprised to find the parts seemingly perfect in form and function. The glands, of course, were largely exposed, but the cicatrix, which I feared would form a constricting band and lead to more or less distortion, was a scarcely discovered white line, while the skin appeared to be just as distensible in its course as anywhere else. No complaint had ever been made, and I am sure none will ever be.

This, Mr. Editor, is a little case, and a very simple one to report in a medical journal, yet it is very practical, and one that involves a highly important member of the human economy. A little carelessness or mismanagement on the part of the medical attendant might have resulted very sadly for the parties interested, while the exercisè of a mordicum of common sense, and perhaps a very little of surgical skill, it may be, has turned the mishap of my little patient to his advantage, for if there be any benefits, hygienic or otherwise, in the Mosaic operation of circumcision, we have them all here, though under a gentile regime and modification, so that in this respect at least, we may possibly regard the little fellow's "latter state as better than his first."

